# Investigation of relaxor and diffuse dielectric phase transitions of Ba_1-X_Bi_x_Ti_0.8_Fe_0·2_O_3_ materials

**DOI:** 10.1016/j.heliyon.2023.e16264

**Published:** 2023-05-13

**Authors:** N. Gouitaa, F.Z. Ahjyaje, T. Lamcharfi, F. Abdi, M. Haddad, M. Sajieddine, M. Ounacer

**Affiliations:** a Signals, Systems and Components Laboratory (LSSC), Electrical Engineering Department, University Sidi Mohamed Ben Abdellah USMBA, FST. Fez, Imouzzer Road B.P. 2202, Morocco; b Spectrometry, Materials and Archaeomaterials Laboratory (LASMAR), University Moulay Ismail Meknès, Morocco; c Physic of Materials Laboratory, FST, University Sultan Moulay Slimane, Beni-Mellal, Morocco

**Keywords:** Ba_1-x_Bi_x_Ti_0.80_Fe_0·20_O_3_ ceramics, Rietveld refinement, RAMAN spectra, Mossbauer, Dielectric properties, Phase transition, Uchino, Cole-cole

## Abstract

Different doping elements have been used to enhance the dielectric properties of BaTiO_3_ ceramic. In this work, the effect of substitution of Ba by Bi in A site and Ti by Fe in B site on structural, dielectric and electrical properties of Ba_1-x_Bi_x_Ti_0.80_Fe_0·20_O_3_ ceramics at (x = 0.00, 0.05, 0.10 and 0.15) was investigated by X-ray diffraction, Raman spectroscopy, Scanning Electron Microscopy (SEM), Mössbauer spectroscopy as well as dielectric measurements. The Rietveld refinement results revealed that the prepared compounds crystallize in both tetragonal (*P*4*mm*) and hexagonal (*P*6_3_/*mmc*) phases for x = 0.00 and 0.05 while at x = 0.10 and 0.15, the hexagonal phase disappears and only the tetragonal phase is fitted. The Raman spectra confirmed the disappearance of hexagonal phase in benefit of tetragonal phase as the Bi^3+^ substitution increases. Based on Mössbauer analyses results, all the samples are in paramagnetic state at room temperature and the Fe is oxidized under Fe^3+^ without the presence of Fe^2+^ or Fe^4+^ ions. The dielectric measurements as function of temperature are studied and tree broad and relaxor phase transitions were detected: from rhombohedral to orthorhombic phase T_R-O_ and to tetragonal ferroelectric phase T_O-T_ then to cubic paraelectric phase T_m_. These phase transitions were displaced to the lower temperature with increasing of Bi^3+^ substitution. The values of ε′_r_ increase gradually with increasing of Bi^3+^contents which confirmed the enhancement of dielectric properties of BaTi_0·80_Fe_0·20_O_3_ by Bi substitution on Ba site. The diffuse phase transitions were described by fitting the modified Uchino relation. The Cole–Cole analyses showed that both the grain and grain boundaries resistivity values are higher for Bi^3+^ substituted samples which are responsible to the dielectric properties improvement.

## 1 Introduction

Multiferroic materials which combine simultaneously ferroelectricity and magnetic order are increasingly attracted the great attention of scientific community over the last decade because of interest in their potential applications in current and emerging technologies at information storage, MERAM, spintronics etc [1,2]. One of the most studied multiferroic material in which coexists the magnetic and electric polarization is BiFeO_3_ (BFO) which is a single-phase magnetoelectric (ME) material. This material has an antiferromagnetic transition at Néel temperature T_N_ = 370 °C [3] and ferroelectric temperature at Curie temperature T_c_ = 830 °C [4,5]. The synthesis of pure BiFeO_3_ is difficult to achieve due to the formation of phases impurities in the Bi_2_O_3_–Fe_2_O_3_ system. The most common impurity phases obtained are Bi_2_Fe_4_O_9_ and Bi_25_FeO_40_ [6,7]. On the other hand, the BFO ceramic has other drawbacks at room temperature which are the high dielectric losses obtained due to the creation of oxygen vacancies and the high leakage current which limit it potential application [8]. So, the goal of the recent studies is to reduce the oxygen vacancies and the leakage current and to enhance the ME coupling coefficient. The same drawback is found for CaCu_3_Ti_4_O_12_ and SrTiO_3_ materials which exhibit a high dielectric losses, and one of the possible solutions found is to co-substitute them on A and B sites [9,10]. The BFO were also substituted by alkaline-earths elements such as: Ba^2+^, Sr^2+^,Ca^2+^ or by rare-earths elements such as: La^3+^, Gd^3+^, Sm^3+^, Dy^3+^ at A site and substituted by non-magneticions Zr^4+^, Ti^4+^, Nb^5+^ at B site [[11], [12], [13], [14], [15], [16], [17], [18], [19], [20], [21], [22], [23]]. Other researchers are oriented to synthesis the nano-composites like BiFeO_3_–BaTiO_3_ [[24], [25], [26], [27]] and it is found a clear decrease in leakage current due to the BaTiO_3_ substitution. However, the BaTiO_3_ (BT) like-perovskite is a suitable lead-free ferroelectric candidate for many applications because its excellent dielectric; ferroelectric and piezoelectric properties [28,29]. To induce a ferromagnetic properties in BT perovskite, it was doped with transition metal element in B site (Ti) such as: Cr, Mn, Fe, Co, Ni, and Cu [30,31] … Various works were reported the enhancement of ferroelectric and magnetic properties of BT doped with, especially, Fe^3+^ ions [32,33]. It is observed that when Fe substituted at B-site (Ti), it improves the coercive field while at A-site (Ba) substitution, the sample saturation magnetization increases [34]. Meanwhile, Fe-doped BaTiO_3_ ceramics show some inconveniences that limit its application which are the low dielectric permittivity values and high dielectric phase transitions, which were confirmed by several works [[35], [36], [37]] On the other hand, the co-substitution of BaTiO_3_ with Fe^3+^ and Bi^3+^ ceramics opens new possibilities to improve physical properties which could be compared with BFO-BT composites properties. The effect of BT co-substituted ceramics with Bi^3+^and Fe^3+^ on structural, dielectric and ferroelectric properties were recently investigated in our works [38,39]. And it has been reported that the substitution of Fe^3+^ions into the Ti^4+^ sites enhance the dielectric permittivity of Ba_0·95_Bi_0·05_TiO_3_ceramic and tree phase transitions were reported assigned to Rhombohedral-Orthorhombic at T_R-O_ = 150 °C, and Orthorhombic-Tetragonal at T_O-T_ = 350 °C then Tetragonal- Cubic at T_m_ > 350 °C. Interestingly, these phase transitions are characterized by a relaxor-type transition and diffuse behavior that were detailed in our previous work [40].

The present work is intended to study the influence of Bi^3+^ doping on structural, dielectric and electrical properties of Ba_1-x_Bi_x_Ti_0.80_Fe_0·20_O_3_solid solution at x = 0.0, 0.05, 0.10 and 0.15 of Bi-doping concentration and to compare the results found with those of BFO-BT composites.

## 2 Experimental method

### 2.1 Synthesis method

**Fig. 1:**
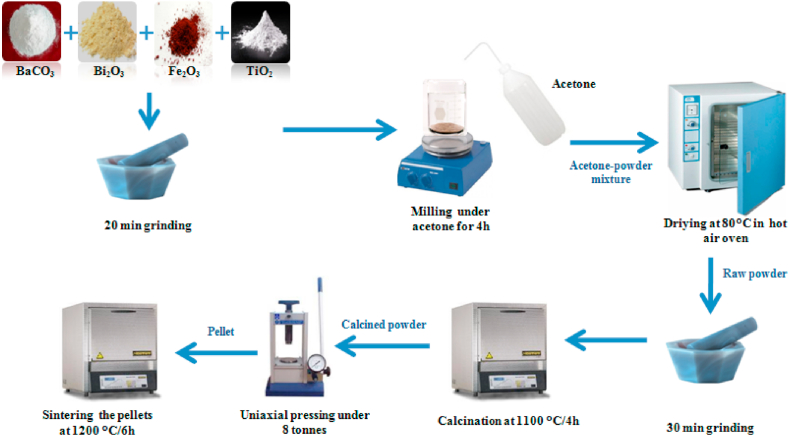
Schematic illustration of Ba_1-x_Bi_x_Ti_0.80_Fe_0·20_O_3_ceramics.

The Ba_1-x_Bi_x_Ti_0.80_Fe_0·20_O_3_ceramics at (x = 0.0, 0.05, 0.10 and 0.15) were prepared by the conventional solid-state method. The high purity precursor (99.9%) of BaCO_3_, Bi_2_O_3_, TiO_2_ and Fe_2_O_3_ were weighted in stoichiometric proportion, grinded for 20 min then milled under acetone for 4 h, as it is shown in Fig. 1. After that the powders were dried at 70 °C for 48 h. The dried powders were grinded using agate mortar for 30 min then they were placed in an alumina nacelle for calcination in air at 1100 °C for 4 h. These calcined powders were mixed with PVA binder (prepared at 2%) and pressed to form the pellets with the diameter and thickness of about 12 mm × 1 mm. The pellets were sintered at 1200 °C/6 h with a heating rate of 3 °C/min. To prepare the pellets for electrical and dielectric measurements, both sides of the pellets were filled by a metal layer.

### 2.2 Materials characterizations

The crystal structure of the calcined materials (Ba_1-x_Bi_x_Ti_0.80_Fe_0·20_O_3_) was characterized by X-ray diffraction (XRD) using Siemens- XPERT-PRO diffractometer with the monochromatic X-ray radiationusing Cu Kα radiation with λ = 1.5406 Å noted within the range of 20–80° with the steep of 0.017°. The refinements of structure were carried out using FULLPROF software based on Rietveld method. The profile refinement parameters included a scale factor, pseudo-Voigt peak. The background was refined to a six-degree polynomial. The lattice parameters, unit cell volume, and the statistical parameters such as: R_p_, R_wp_ and χ^2^ were obtained using Fullprof refinement software. The valence of Fe was analyzed withthe^57^Fe Mössbauer spectra at room temperature and all the values of isomer shift within this paper are related to the α-Fe standard. The Raman spectroscopy was successfully used to study the structural evolution of ceramics, and measurements were performed at room temperature in the region of 100–1000 cm^−1^. The grain size and the micromorphology were detected on a fracture of surface by a scanning electron microscope (SEM). And the dielectric properties as function of frequency and temperature were studied with Agilent E4980A (20 Hz-2MHz) under 1 V of oscillatingvoltage. The obtained results provide the capacitance (C_p_) values which are converted to the dielectric permittivity. The imaginary and real parts of the impedance were also calculated to plot the Nyquistdiagram. The obtained Nyquist plots were fitted using the software Z-view to study the grain and grains boundaries contribution in the prepared samples.

## 3 Results and discussion

### 3.1 Crystal phase analyses

**Fig. 2:**
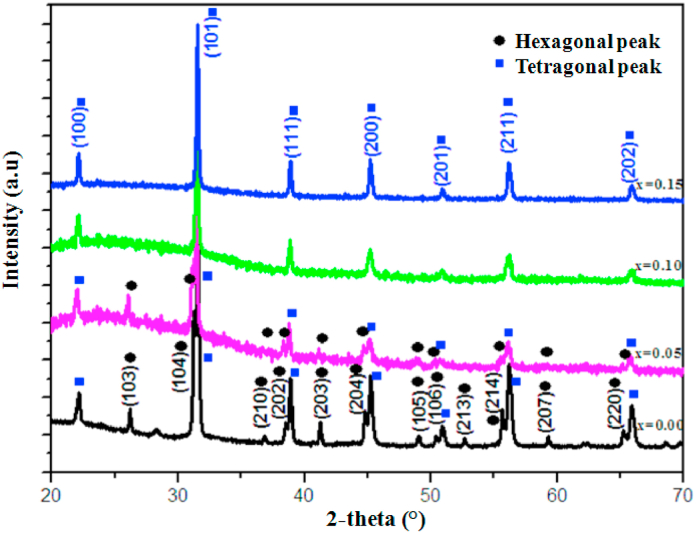
XRD spectra of Ba_1-x_Bi_x_Ti_0.80_Fe_0·20_O_3_ ceramics (x = 0.00, 0.05, 0.10 and 0.15).

**Fig. 3:**
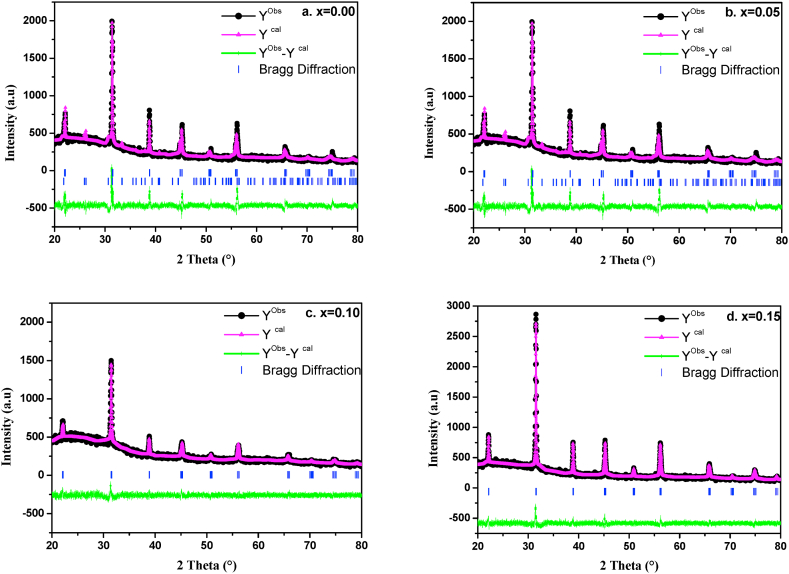
Rietveld plots of XRD data for Ba_1-x_Bi_x_Ti_0.80_Fe_0·20_O_3_ceramics for x = a. 0.00, b. 0.05, c. 0.10 and d. 0.15. The points are the observed profile while the solid line is the calculated profile. Positions for the Bragg reflection are marked by vertical bras. The line curve at the bottom of the diagram gives the difference between observed and calculated profile.

Fig. 2 shows the **XRD patterns of** Ba_1-x_Bi_x_Ti_0.80_Fe_0·20_O_3_ ceramics for compositions x = 0.00, 0.05, 0.10 and 0.15 calcined at 1100 °C/4 h. For compositions with x = 0.00 and 0.05, the diffraction peaks were identified with coexistence of two tetragonal and hexagonal phases. Above 0.10 of Bi contents, the hexagonal peaks disappeared and only the tetragonal phase is identified. To study the effect of Bi^3+^ on the crystal structure of BaTi_0·80_Fe_0·20_O_3_ ceramic, a profile refinement of the XRD data was performed. The **Rietveld refinement results** of Ba_1-x_Bi_x_Ti_0.80_Fe_0·20_O_3_ compounds are shown in Fig. 3. From these results of refinement all the diffraction peaks are fitted with the coexistence of tetragonal and hexagonal phases with *P*4*mm* and P63 mmc space group respectively for prepared samples at x = 0.00 and 0.05 (Fig. 3 a. And b). While at x = 0.10 and 0.15 (Fig. 3c and d) only the tetragonal phase is fitted. Indeed, with increasing of Bi contents, the tetragonal phase becomes predominant at percentage higher than 0.10 of Bi substitution. Thus suggesting that the concentration higher than 0.10 of Bi removes the hexagonal phase formation which is occurred in BT ceramic by Fe^3+^ substitution [36]. According to the refinement, the Bi^3+^ and Fe^3+^ are incorporated in BaTiO_3_, so there is no secondary phase observed in diffraction patterns. Contrary to the results found by P. Esther Rubavathi et al. [41], for BFO-BT composites which reported the appearance of secondary phases of Bi_2_Fe_4_O_9_ and Bi_2_FeO_40_ related to the slow kinetics formation of BiFeO_3_.

**Fig. 4:**
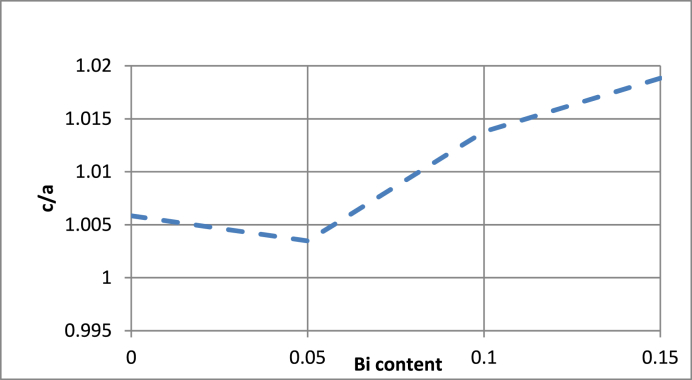
Evolution of tetragonality c/a of Ba_1-x_Bi_x_Ti_0.80_Fe_0·20_O_3_ ceramics as function of Bi substitution from x = 0.00 to 0.15.

**The Rietveld** parameters of Ba_1-x_Bi_x_Ti_0.80_Fe_0·20_O_3_ ceramics are carefully determined by FULLPROF program which are listed in Table .1. A good agreement between the observed and calculated patterns was obtained with R_p_ ≤ 8.31%, R_wp_≤13.4% and chi-squared (χ^2^)≤3.90 for all ceramics which is in good agreement with the value reported in literature [42]. From Fig. 4 the calculated value of tetragonality of these ceramics is found to increase with increasing of Bi content confirming the occurrence of phase transition from hexagonal to tetragonal phase. In addition, the cell volume is found to decrease which is probably due to the substitution of Ba^2+^with ionic radius (r_i_ (Ba^2+^) = 1.35 Å) which is bigger than that of Bi^3+^ (r_i_ (Bi^3+^) = 1.2 Å). In these ceramics, we note the creation of oxygen vacancies when the Bi^3+^ substitute the Ba^2+^ ions to compensate to charge valence. Hence, the distortion observed in these ceramics could also be due to the creation of oxygen vacancies.

**Table 1:** Refined structural parameters of Ba_1-x_Bi_x_Ti_0.80_Fe_0·20_O_3_ ceramics for x = 0.00, 0.05, 0.10 and 0.15.

x	0.00		0.05		0.10	0.15
Statistical parameters	Tetragonal	Hexagonal	Tetragonal	Hexagonal	Tetragonal	Tetragonal
	R_p_ = 6.39	R_p_ = 8.31	R_p_ = 5.96	R_p_ = 7.55	R_p_ = 4.78	R_p_ = 6.71
	R_wp_ = 8.54	R_wp_ = 13.40	R_wp_ = 7.53	R_wp_ = 11.4	R_wp_ = 6.11	R_wp_ = 8.66
	χ^2^ = 1	χ^2^ = 3.90	χ^2^ = 1.34	χ^2^ = 3.54	χ^2^ = 0.960	χ^2^ = 1.56
Bragg R-factor	55.13	36.02	36.5	40.6	21.8	14.6
Lattice constants (Å)	a = b = 4.0067	a = b = 5.839	a = b = 4.0052	a = b = 5.654	a = b = 4.0047	a = b = 4.0056
	c = 4.03009	c = 13.7607	c = 4.01916	c = 13.423	c = 4.05988	c = 4.081004
Volume (Å^3^)	64.697	406.309	64.475	420.19	63.54	62.86
Space group	P 4 m m	P 63 m m c	P 4 m m	P 63 m m c	P 4 m m	P 4 m m

### 3.2 Raman spectra

**Fig. 5:**
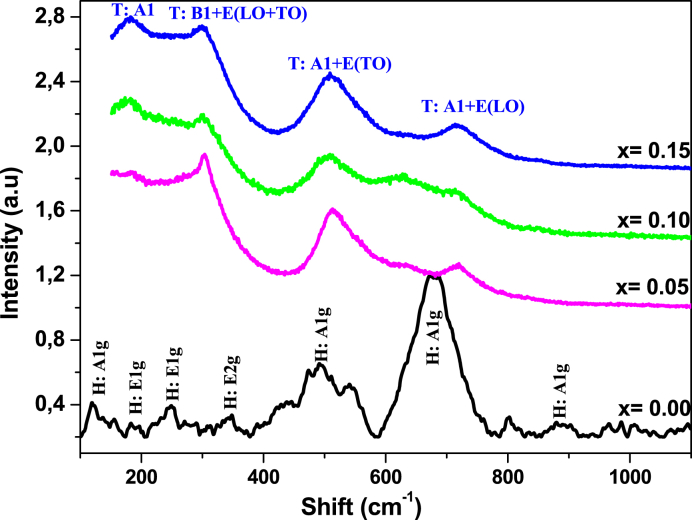
Raman spectra of Ba_1-x_Bi_x_Ti_0.80_Fe_0·20_O_3_ceramics at different substitution concentrations measured at room temperature.

The Raman spectroscopy is a sensitive method to detect the short range ordering structure and can identify local and static symmetries. Fig. 5 shows the Raman spectra of Ba_1-x_Bi_x_Ti_0.80_Fe_0·20_O_3_ceramics for x = 0.00 to 0.015 of Bi content. For x = 0.00 (BTFO), the seven bands at: 115.5, 155,269.5, 367, 422.5, 588 and 738 cm^−1^, According to Murugesan et al. [43] and other researchers [44], are attributed to A_1g_, E_1g_, E_1g_, E_2g_, A_1g_, A_1g_ and A_1g_ respectively and matched with the typical Raman peaks of h-BaTiO_3_ which confirm that the hexagonal phase is predominant at x = 0.00. With Bi substitution at x = 0.05, we notice the appearance of intense bands around 266 and 520 cm^−1^ assigned to the A_1_ (TO) and A_1_+E (TO) modes associated to Ba site and T_i_–_O_ chains [45] respectively. Whereas the broad and asymmetric band around 309 cm^−1^ assigned to the B_1_+E (LO + TO) mode, attests the tetragonal phase formation [46,47]. Finally, the broad peak at 720 cm^−1^ is attributed to A_1_+E (LO) mode. It is clear that all the tetragonal peaks are more dominant and became sharp and intense with the increase of Bi content indicating the increase in tetragonality of theses samples. On the other hand, the A_1g_ band around 738 cm^−1^ which represents the hexagonal phase is not dominant, become weaker and finally disappear completely for x = 0.10 and 0.15 of Bi content indicating that the hexagonal phase is not dominant for the substitution ceramics. The same results are found by Rietveld refinement results.

### 3.3 Morphological analyses

**Fig. 6:**
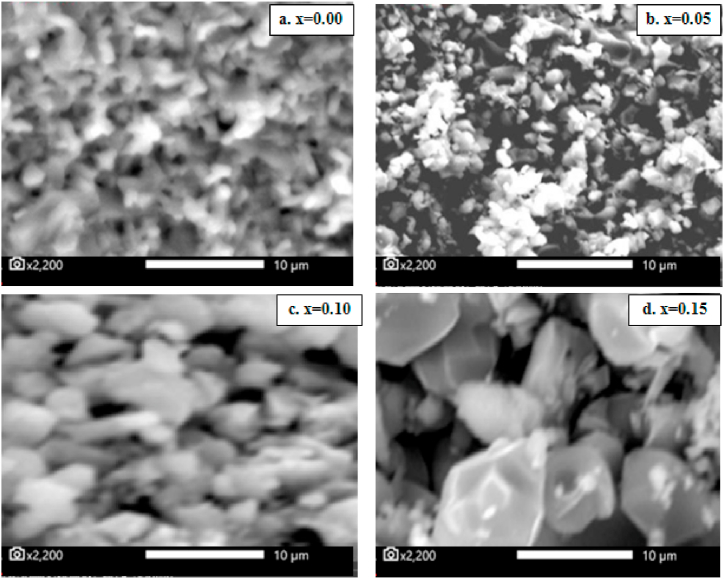
SEM micrographs of Ba_1-x_Bi_x_Ti_0.80_Fe_0·20_O_3_ ceramics at x = a. 0.00,b. 0.05, c. 0.10 and d. 0.15.

Fig. 6 shows the SEM micrographs of Ba_1-x_Bi_x_Ti_0.80_Fe_0·20_O_3_ pellets for x = 0.00, 0.05, 0.10 and 0.15 sintered at 1200 °C for 6 h. It is clear that the microstructure and grain growth change with Bi substitution. The samples at x ≤ 0.05 of Bi content exhibit fine grains with non-homogenous grains shape. The size distribution of the formed grains is found to be non-uniform which may be reflecting the coexistence of two phases in these materials. The grain form becomes more homogeneous with doping of Bi at x = 0.15 comparing with the others samples which grain forms are inhomogeneous especially at x = 0.00. Moreover, the shape of grains is transformed to quadratic for x = 0.15 of Bi sample related to the dominance of quadratic phase found in XRD and Raman results. The average grains size, calculated from FESEM images, is found to be 1.478 μm, 1.896 μm, 1.990 μm and 3.188 μm, respectively, for x = 0.00, 0.05, 0.10 and 0.15. It is clear that the average grain size increases with increasing of Bi content.

### 3.4 Mössbauer analysis

**Fig. 7:**
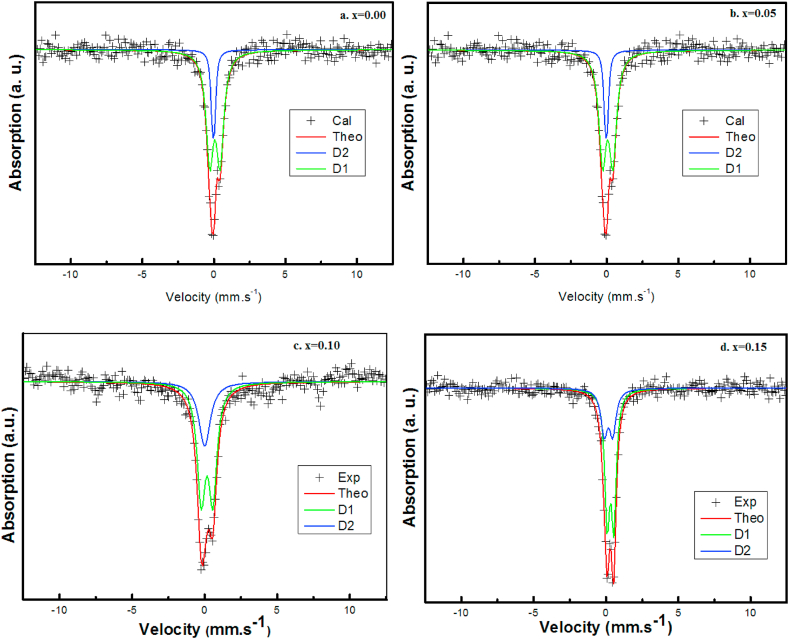
Mössbauer spectra of ^57^Fe of Ba_1-x_Bi_x_Ti_0.80_Fe_0·20_O_3_ ceramics for x = a. 0.00, b. 0.05, c. 0.10 and d. 0.15.

Mössbauer spectroscopy is widely used to identify the electronic state of the active metal, namely iron in our ceramics. The Mössbauer spectra of ^57^Fe for Ba_1- x_Bi_x_Ti_0.80_Fe_0·20_O_3_ ceramics (x = 0.00, 0.05, 0.10 and 0.15) at room temperature are shown in Fig. 7. All the spectra show the presence of a doublet and not a sextuplet, which characterizes the paramagnetic state of these ceramics. The hyperfine parameters deduced after adjustment of the experimental spectra are grouped in Table 2. Based on the isomer shift (IS) values, a parameter sensitive to the iron valence and the geometry of the coordinating anions, which are less than 0.42 characteristic of +3 valence of Fe state in an octahedral oxygen environment that exclude the existence of Fe^2+^ or Fe^4+^ [48].

**Table 2:** Hyperfine parameters of Ba_1-x_Bi_x_Ti_0.80_Fe_0·20_O_3_ ceramics.

x	Doublets	Area	IS (mm.s^−1^)	QS (mm.s^−1^)	FWHM
0.00	Doublet-1	78	0.16	0.34	0.22
	Doublet-2	22	0.08	0.08	0.24
0.05	Doublet-1	74.80	0.24	0.4	0.28
	Doublet-2	25.20	0.14	0.10	0.13
0.10	Doublet-1	31.20	0.28	0.30	0.27
	Doublet-2	68.80	0.42	0.23	0.20
0.15	Doublet-1	71.10	0.27	0.40	0.33
	Doublet-2	28.90	0.10	0.01	0.47

All the Mössbauer spectra present two octahedral types which are D_1_ octahedron and D_2_ octahedron. The first doublet D_1_ corresponds to the pentahedron Fe^3+^ while the second doublet D_2_ is attributed to the octahedral Fe^3+^ [49]. Both octahedral and pentahedron sites present a low symmetry for x = 0.10 because its quadrupole separation coefficient (QS) increases. Whereas at x = 0.15 of Bi content, the QS value is very close to zero which confirm the high quadtratic symmetry of this sample. On the other hand, the IS parameter related to Fe^3+^ octahedral decreases for x = 0.15 of Bi substitution indicating an increase of s density of electron which is caused by the favor creation of oxygen vacancies at this concentration [50]. However, the charge difference between Ba^2+^ and Bi^3+^ ions promotes the creation of oxygen vacancies at high level of Bi (x = 0.15), in octahedral site [51] which is the main reason for the decrease of IS at this content.

### 3.5 Dielectric properties

#### 3.5.1 Dielectric properties versus temperature

**Fig. 8:**
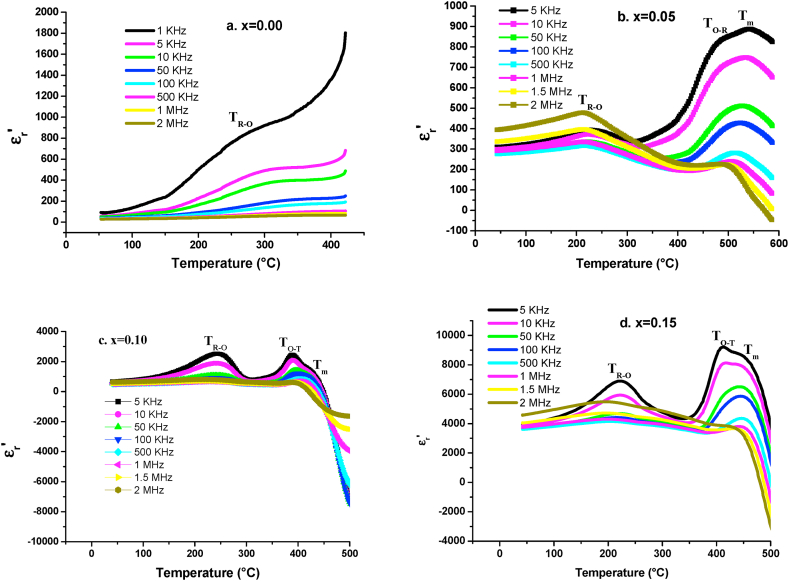
Dielectric constant dependent temperature at different frequencies of Ba_1-x_Bi_x_Ti_0.80_Fe_0·20_O_3_ceramics for x = a. 0.00, b. 0.05, c. 0.10 and d. 0.15.

**Fig. 9:**
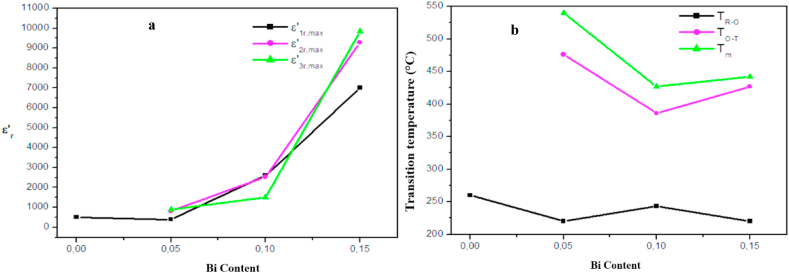
**a**. Evolution of maximum of dielectric constant as function of Bi content from x = 0.00 to 0.15. **b.** Evolution of temperature of phase transition as function of Bi content from x = 0.00 to 0.15.

Fig. 8 displays the evolution of the real part of the dielectric permittivity (ε′_r_) as a function of temperature (from R.T to 450 °C) at various frequencies (from 5 KHz to 2 MHz). We can notice in Fig. 8 a, one phase transition from rhombohedral to orthorhombic phase T_R-O_. While, there are tree dielectric anomalies for all the co-substituted samples (Fig. 8b, c and d). These tree peaks are due to different phase transitions of BiFeO_3_ and BaTiO_3_ [52]. Based on our previous works [39,40] and in accordance with the literature [52,53], these peaks are corresponding to the phase transition from rhombohedral to orthorhombic phase T_R-O_ at low temperature and to tetragonal ferroelectric phase T_O-T_ at intermediate temperature then to a cubic paraelectric phase T_m_ at high temperature. The evolution of dielectric permittivity and transition temperature of these tree phase transitions at 5 KHz are represented in Fig. 9. As it is shown, the ε′_r_ values of BBTFO ceramics increase significantly with Bi doping for all phase transitions (Fig. 9a) and reach a maximum of 7002, 9281 and 9854 for T_R-O_, T_O-T_ and T_m_ respectively, at x = 0.15 of Bi content (Table 3). Indeed, with the increase of the charge valence from +2 of Ba to +3 of Bi on A site, a residual positive charge appears and the mutual effect between A and B sites (Ti^4+^) becomes stronger. Thus, the polarization of Ti^4+^ is improved, then the dielectric constant increases sharply [54,55]. This increase of ε′_r_ with increasing Bi is also found by S. Islam et al. [56], for Bi-substituted BaTiO_3_ ceramics, but the values of ε′_r_ they obtained are larger than our values. This difference is caused by the substitution of BaTiO_3_ by Fe in the Ti site which has the effect of decreasing ε′_r_ [37]. Moreover, it is noticed that the phase transition temperatures decrease with the Bi substitution (Fig. 9b). However, F. BAHRI et al. [57] have reported that the value of the transition temperature of Ba_1-x_Bi_2x/3_TiO_3_ ceramics decreases when x increases for 0 <x < 0.15. And they attributed this decrease to the combination of two factors, namely the replacement of the large Ba^2+^ cation by the smaller Bi^3+^ (r_Ba2+_ = 1.35 Å, r_Bi3+_ = 1.2 Å) and the creation of cation vacancies. These results agree with our results. So, the goal of the co-substitution of BTF by Bi ions in A-site which is the increase in the value of the dielectric permittivity and the decrease in the phase transition temperature is succeeded.

**Table 3:** ε_r,max_ and T_m_ values of the tree phase transitions.

x	0.00	0.05	0.10	0.15
ε_**1r,max**_	831	393	2600	7009
ε_**2r,max**_	–	812	2530	9281
ε_**3r,max**_	–	896	1502	9854
**T**_**R-O**_**(°C)**	270	220	243	220
**T**_**O-T**_**(°C)**	–	476	386	410
**T**_**m**_**(°C)**	–	540	427	442

#### 3.5.2 Diffuse and relaxor phenomena of the three phase transitions

The tree phase transitions show a strong diffuse behavior for all the compositions. The T_O-C_ phase transition in our samples present a relaxor behavior due to the relaxation process associated with defect dipoles such as the oxygen-vacancy and the valence fluctuation.

The degree of relaxor behavior could be estimated by the empirical parameter:

ΔT_relax_ = T(100 kHz) - T (5 kHz)

Where T (100 kHz) and T (5 kHz) are temperature corresponding to the dielectric constant maximum at frequencies of 100 KHz and 5 kHz, respectively, selected based on the minimum frequency (5 KHz) and the maximum frequency in which the phase transition is observed (100 KHz). The value of this parameter for all the samples is calculated and reported in Table 4. As it is observed, all the values of ΔT_relax_ are different to zero for all the samples which confirm the relaxor behavior in our ceramics. Otherwise, the ΔT_relax_ is maximal for T_m_ at the sample x = 0.01. In perovskite-like compounds, the relaxation behavior appears when at least two cations occupy the same A or B crystallographic site. In our composition, the Bi^3+^ atom occupies the A site and creates sites vacancies on the Ti^4+^: V_Ti_ site due to its valence which is higher than that of A-site atom (Ba^2+^). Moreover, the Fe^3+^ atom occupies the Ti^4+^ site and thus creates sites vacancies on the Ba^2+^ site: V_Ba_. Therefore, the difference of the valence in the site of Ba with Bi as well as in the site of Ti with Fe creates more disorder in the ceramic site of BaTiO_3_ and thus generates a dielectric constant dependent on frequency and temperature behavior called relaxation [[58], [59], [60]].

**Table 4:** ΔT_relax_, γ and δ values for Ba_1-x_Bi_x_Ti_0.80_Fe_0·20_O_3_ ceramics.

Transition	parameter	0.00	0.05	0.10	0.15
T_R-O_	**ΔT**_**relax**_**(°C)**	25	7	3	2
	**δ**	Very board	86.17	29.22	56.7
	**γ**	Very board	1.49	2.00	2.00
T_O-T_	**ΔT**_**relax**_**(°C)**	–	30	6	20
	**δ**	–	Very board	22.9	61
	**γ**	–	Very board	2.28	1.99
T_m_	**ΔT**_**relax**_**(°C)**	–	10	20	12
	**δ**	–	323	12.18	82.82
	**γ**	–	1.08	1.62	1.01

The diffuseness character of the tree phase transitions, degree of diffuseness or the diffuseness coefficient (γ) was obtained by fitting the dielectric constant curves with the modified Uchino's phase transitions as given below [61]:

$$1/\varepsilon ’r=1/\varepsilon ’r,\max\left[\;1+{\left(\frac{\left(T-T_{m}\right)}{2\delta }\right)}^{\gamma }\right]$$

This equation may be written as the following:

Ln [((ε′_r,max_/ ε′_r_)-1)] = γ ln (T-T_m_) - γ ln2δ

**Fig. 10:**
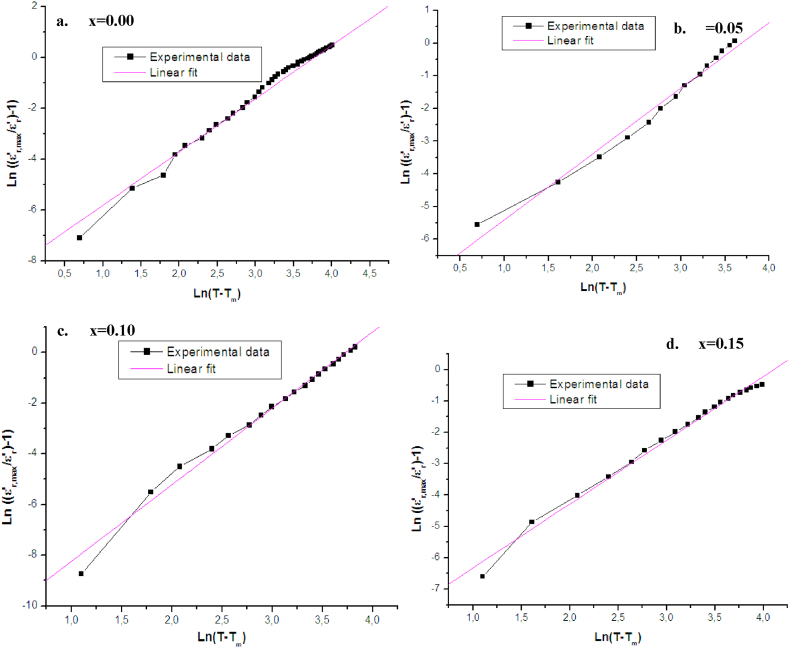
The plot of Ln (1/ε′_r_ - 1/ε′_r,max_) as a function of Ln (T-T_m_) at 5 KHz for Ba_1-x_Bi_x_Ti_0.80_Fe_0·20_O_3_ceramics for x = a. 0.00, b.0.05, c. 0.10 and d.0.15.

Where T_m_ is the temperature corresponding to the maximum of dielectric constantof T_T-O_,ε′_r,max_ is the permittivity at T_m_, γ is the degree of dielectric relaxation. It value is 1 for normal ferroelectrics following Curiee Weiss law, 2 for ideal relaxor ferroelectrics. In general γ takes a value between these limits (1 <γ < 2) indicating an incomplete diffuse phase transition. The value of δ represents the degree of diffuseness for transition peaks. Linear relationships are observed in the plot of ln ((ε′_r,max_/ε′_r_)-1)versus ln (T-T_m_) at 5 KHz frequency for all the samples as it is shown in Fig. 10. By fitting the experimental data to the modified Uchino equation, the values of γ and δ are listed in Table 4.

We can notice that certain values of the diffusivity parameter γ are close to 1 but the transition remains always diffuse with a relaxor character, explained by the large values of δ. While other values of γ are between 1.49 and 2 indicating a relatively diffuse and relaxor type of transition. On the other hand, γ values are greater than 2 or much less than 1 indicating that the distribution of the phase temperatures of these ceramics is no longer a Gaussian as it was described by G.A. Smolenski [62] which limits the validity of the Uchino law of these compounds. We note also that the diffusion factor δ is maximum at x = 0.05 for the three phase transitions and decreases at x = 0.10 then it increases at x = 0.15 of Bi content. The maximum value of δ shows that the three phase transitions are highly broad at x = 0.05, and less broad at x = 0.10. The diffuse phase transition behavior as observed in these ceramics can be induced by multiple reasons such as the variation of the composition, the micropolar regions or a coupling of the local order and disorder parameter generated by a local constraint. The disorder in the structure of Ba_1-x_Bi_x_Ti_0.80_Fe_0·20_O_3_ ceramics may arise from the substitution of two Bi^3+^ and Fe^3+^ ions in two different crystallographic sites Ba^2+^ and Ti^4+^ respectively, thus leading to nanometric heterogeneity in the compounds and, consequently, to a distribution of various local Curie points [63,64], then to a random distribution of the electric strain field in mixed oxide compounds which is the main reason leading to the scattering behavior as reported by B.E. Vugmeister [65].

### 3.6 Cole-cole analyses

**Fig. 11:**
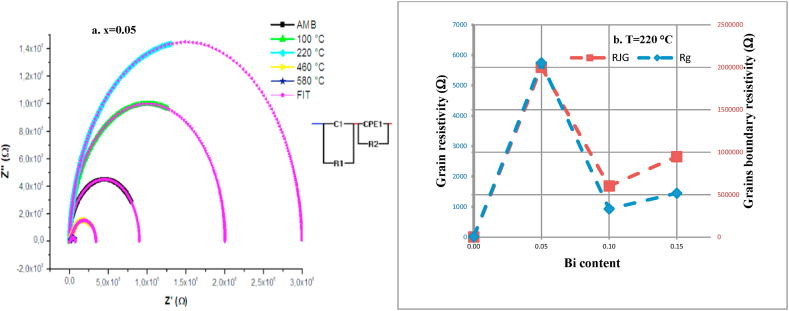
**a.** Complex impedance spectrum of Ba_1-x_Bi_x_Ti_0.80_Fe_0·20_O_3_ceramic at x = 0.05 at different temperature. **b.** Evolution of grains and grains boundaries resistivity of Ba_1-x_Bi_x_Ti_0.80_Fe_0·20_O_3_ ceramics for x = 0.00, 0.05, 0.10 and 0.15 measured at 220 °C.

To understand the high-temperature dielectric dispersion, the complex Cole–Cole equation was used to analyze the impedance data. Fig. 11 a. shows the complex impedance spectrum plots of Ba_1-x_Bi_x_Ti_0.80_Fe_0·20_O_3_ ceramics for x = 0.05 at different measurement temperature from 100 °C to 400 °C. The experimental data is simulated using the electrical equivalent circuit R//C and R//CPE connected in series as it is shown inset of Fig. 11 a.The slope of the curves showed a tendency to bend towards the abscissa to form a single semi-circle for each temperature. This single semi-circle gives evidence to the contribution of grains boundaries rsistance and the right extremity of the semicircle corresponds to the grain resistance value [66]. On the other hand, the centers of semicircles are above the real axe of Z’ which confirmed that the relaxation in these samples is deviated from the non-debye type [67,68]. The radius of curvature of the arcs increases with increasing temperature from ambient to 220 °C, which reveals that the conductivity of the sample decreases as temperature increases and indicate also a positive temperature coefficient of resistivity (PTCR) behavior of the test materials [69,70]. Above 220 °C, we notice a clear increase in radius of semi-arcs indicating a negative temperature coefficient of resistivity (NTCR). The values of both grain and grains boundaries resistances are extracted from the fit of the test materials by using the equivalent circuit and it variation depending on Bi content, at fixed temperature of 220 °C, was plotted in Fig. 11 b.According to the values found, it is clear that the grains boundaries are more resistive than grains. This may be related to the occurrence of dangling bonds at grain boundaries and a Schottky-like electrical barrier between grains and grain boundaries [71]. We note also, that the grains and grains boundaries have the same evolution with Bi substitution. Indeed, both the resistance of grains and grains boundaries of substituted samples with Bi are higher than that of un-substituted samples. This increase in resistivity values is probably the origin of enhancement of dielectric properties.

## 4 Conclusion

In this study the effect of Bi substitution on structural and dielectric properties of BaTi_0.80_Fe_0·20_O_3_ceramics at x = 0.00, 0.05, 0.10 and 0.15 was studied. The Rietveld refinement showed the formation of tetragonal and hexagonal phase for x = 0.00 and 0.05 while only the tetragonal phase is fitted for x = 0.10 and 0.15 of Bi content. This phase change is confirmed by Raman spectra. The SEM micrographs revealed a change of grains shape from semi-circular to quadratic shape with the increasing of Bi content. The dielectric measurement showed the existence of tree phase transitions. The temperature corresponding to these phase transition shifted to lower temperature and ε′_r_ values are enhanced with Bi substitution. The relaxation behavior present in all the samples is related to the defect dipoles such as the oxygen-vacancy and the valence fluctuation of Fe ions (between Fe^3+^ and Fe^2+^) during the heat treatment. The diffuseness character is defined by modified Ushino law and indicates that the diffusivity γ corresponds to a very broad relaxation and a high disorder which is more pronounced for the sample at x = 0.05. The complex impedance spectroscopy approved the grain and grain boundary contributions which explain the enhancement of dielectric properties in these samples. These dielectric properties obtained make Ba_1-x_Bi_x_Ti_0.80_Fe_0·20_O_3_ a potential candidate for dielectric and electrical devices. In the next works, because the new discovery of these materials, the magnetic properties should be studied taking into account the thickness and density of grains.

## Author contribution statement

N. Gouitaa: Conceived and designed the experiments; Performed the experiments; Analyzed and interpreted the data; Wrote the paper.

F. Z. Ahjyaje: Conceived and designed the experiments; Contributed reagents, materials, analysis tools or data; Wrote the paper.

T. Lamcharfi, F. Abdi, M. Haddad, M. Sajieddine, M. Ounacer: Conceived and designed the experiments; Analyzed and interpreted data; Contributed reagents, materials, analysis tools or data; Wrote the paper.

## Data availability statement

Data included in article/supp. Material/referenced in article.

## Declaration of competing interest

The authors declare that they have no known competing financial interests or personal relationships that could have appeared to influence the work reported in this paper
